# Biological Activities of Stilbenoids

**DOI:** 10.3390/ijms19030792

**Published:** 2018-03-09

**Authors:** Bolanle C. Akinwumi, Kimberly-Ann M. Bordun, Hope D. Anderson

**Affiliations:** 1 College of Pharmacy, Rady Faculty of Health Sciences, University of Manitoba, 750 McDermot Avenue, Winnipeg, MB R3E 0T5, Canada; handerson@sbrc.ca; 2 Canadian Centre for Agri-Food Research in Health and Medicine, St. Boniface Hospital Research Centre, 351 Taché Avenue, Winnipeg, MB R2H 2A6, Canada; kbordun@sbrc.ca; 3 Department of Pharmacology and Therapeutics, Max Rady College of Medicine, University of Manitoba, 753 McDermot Avenue, Winnipeg, MB R3E 0T6, Canada

**Keywords:** stilbenoid phenolics, resveratrol, pterostilbene, gnetol, cardioprotective, biological activities

## Abstract

Stilbenoids are a group of naturally occurring phenolic compounds found in various plant species. They share a common backbone structure known as stilbene, but differ in the nature and position of substituents. Stilbenoids are classified as phytoalexins, which are antimicrobial compounds produced de novo in plants to protect against fungal infection and toxins. In this review, the biological effects of stilbenoids such as resveratrol, pterostilbene, gnetol and piceatannol are discussed. Stilbenoids exert various biological activities ranging from cardioprotection, neuroprotection, anti-diabetic properties, depigmentation, anti-inflammation, cancer prevention and treatment. The results presented cover a myriad of models, from cell culture to animal studies as well as clinical human trials. Although positive results were obtained in most cell culture and animal studies, further human studies are needed to substantiate beneficial effects of stilbenoids. Resveratrol remains the most widely studied stilbenoid. However, there is limited information regarding the potential of less common stilbenoids. Therefore, further research is warranted to evaluate the salutary effects of various stilbenoids.

## 1. Introduction

**Figure 1:**
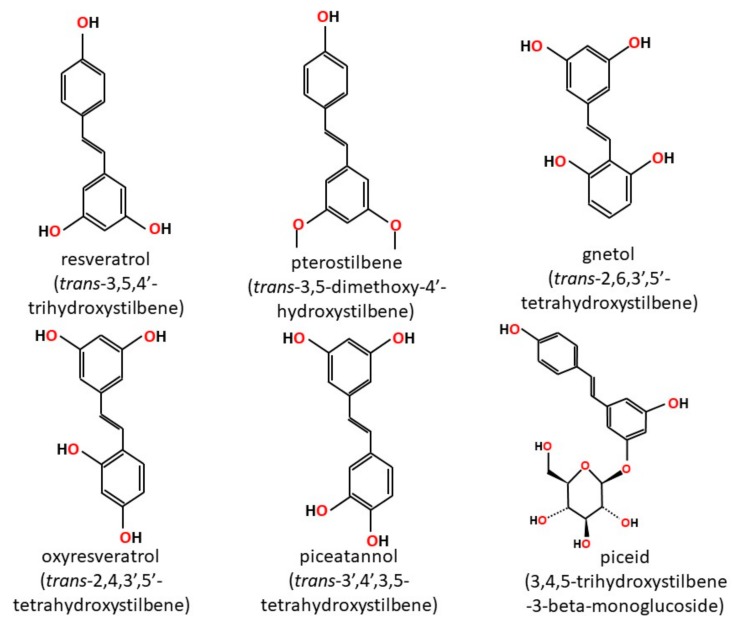
Chemical structures of selected stilbenoid phenolics showing common stilbene backbone.

Stilbenoids are a group of naturally occurring phenolic compounds found in various plant species [1]. They share a common backbone stilbene structure but differ in the type and position of substituents on the ring (Figure 1). Stilbenoids exist as monomers or oligomers. They may also be found free (aglycone) or conjugated as glucosides. For example, piceid is resveratrol-3-*O*-glucoside [2,3]. The monomeric stilbene (*trans*-1,2-diphenylethylene) aglycone structure consists of two phenyl rings joined by an ethylene bridge. Stilbenes may exist as the *cis*- or *trans*-isomer, but the *trans*-isomer is the more common and stable configuration [1]. 

**Figure 2:**
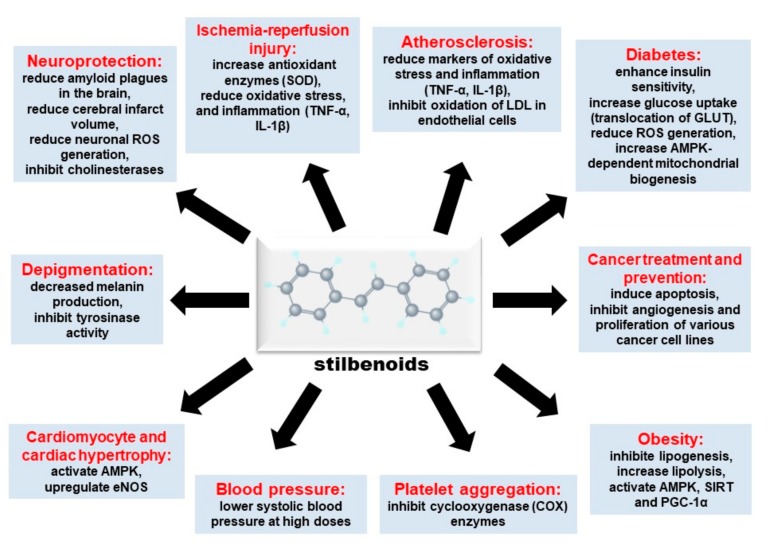
Summary of mechanisms of biological activities of stilbenoids.

Stilbenes are synthesized in plants via the phenylpropanoid pathway in a similar fashion to flavonoids [1]. The biosynthetic pathway begins with the conversion of phenylalanine to cinnamate by the action of phenylalanine ammonia lyase [4]. An acetyl-CoA group is thereafter added to cinnamate by the enzyme, CoA ligase, to form cinnamoyl-CoA. Lastly, cinnamoyl-CoA is converted to stilbenoids by the action of stilbene synthase in a process that utilizes three units of malonyl CoA [4]. Stilbenoids can be further processed by methylation, glucosylation and prenylation. The most popular and perhaps the most widely studied stilbenoid is resveratrol. There are other structural analogs with potentially beneficial medicinal properties, but the information on those biological effects is limited. The biological activities of stilbenoids reviewed in this paper and the respective underlying mechanisms are summarized in Figure 2 below.

## 2. Stilbenoids

Stilbenoids are phytoalexins, which are antimicrobial compounds produced de novo to protect the plant from fungal infection and toxins [5,6]. Resveratrol (*trans*-3,5,4′-trihydroxystilbene) is found in *Vitis* species (grapes), red wine and other plant species [5,7]. Pterostilbene (*trans*-3,5-dimethoxy-4′-hydroxystilbene) is a dimethyl ether analog of resveratrol and is found in plant species such as *Pterocarpus marsupium* [8], *Vitis* [6], and *Vaccinium* species (blueberries) [9]. HPLC analysis revealed that resveratrol and pterostilbene are present in drakshasava, an ancient cardiotonic grape preparation used in Ayurvedic medicine [10].

Gnetol (*trans*-2,6,3′,5′-tetrahydroxystilbene) is another stilbenoid found in several species of the genus *Gnetum*. *Gnetum* is a gymnospermous plant with more than 35 species that occur as trees, shrubs and lianas. Specifically, gnetol has been isolated from *Gnetum ula* [11], *G. gnemon* [12], *G. montanum* [13], *G. klossii* [14] and *G. hainanese* [15]. The seeds and leaves of *G. gnemon*, also known as melinjo, are eaten as vegetables in Indonesia [16]. Gnetol is used in folk medicine for arthritis and asthma [17].

Piceatannol (*trans*-3′,4′,3,5-tetrahydroxystilbene) is commonly found in berries, grapes, rhubarb (*Rheum* species), passion fruit (*Passiflora* species) and white tea [18]. Piceatannol is a metabolite of resveratrol, produced by the enzyme CYP1B1 in humans [19]. Thus, resveratrol is considered a pro-drug for piceatannol. Due to the presence of an additional hydroxyl group and subsequent formation of a semiquinone radical, piceatannol exhibits more powerful antioxidant activity compared to resveratrol [20,21]. Another stilbenoid is oxyresveratrol, an isomer of hydroxylated resveratrol. Oxyresveratrol is found in the bark of *Morus alba* [22], and in the heartwood of *Artocarpus lakoocha* [23]. Oxyresveratrol exerts its effects primarily through the inhibition of tyrosinase, an enzyme which is responsible for the pigmentation found in skin, eyes and hair [22].

### 2.1. Bioavailability

Resveratrol exhibits poor water solubility (<0.05 mg/mL) and low oral bioavailability [24]. Complexation with cyclodextrins improves aqueous solubility of resveratrol but not bioavailability [24]. Administration of higher doses of resveratrol also did not improve the pharmacokinetic profile [24]. Other attempts to improve bioavailability of stilbenoids include complexation with bile acids [25], incorporation into liposomes [26] and formulation into nanoparticle delivery systems [27,28]. 

The bioavailabilities and half-lives of selected stilbenoids following oral administration in rats are shown in Table 1. Pterostilbene exhibited the highest bioavailability of 80% [29] while gnetol exhibited the lowest oral bioavailability of 6.59% [30]. After intravenous administration, resveratrol exhibited a very short half-life of 14 min due to rapid metabolism. The reported oral bioavailability values for resveratrol range from 20% to 29.8% [29,31]. Although gnetol has the lowest bioavailability, the reported half-life after oral administration of 100 mg/kg in rat was 4.2 h [30]. This value is significantly longer than values reported for resveratrol (1.48 h) [31] and pterostilbene (1.73 h) [32]. Moreover, higher levels of the gnetol glucuronide metabolite persisted for up to 72 h in serum after oral administration [30]. It is possible that gnetol glucuronide may be reconverted into free gnetol and thus compensate for the low bioavailability. 

The presence of two methoxy groups in the pterostilbene structure makes it more lipophilic and thus more bioavailable [29]. Pterostilbene is also more metabolically stable because it has only one free hydroxyl group available for glucuronidation or sulphation. Indeed, an enzyme kinetic glucuronidation assay performed in human liver microsomes showed that resveratrol was more efficiently metabolized by glucuronidation compared to pterostilbene [36]. This may further contribute to higher levels of the free pterostilbene and higher bioavailability. Thus, based on the pharmacokinetic profile of stilbenoids, pterostilbene is more bioavailable than resveratrol and, as such, may be a potential candidate as an alternative to resveratrol.

### 2.2. Metabolism

The low bioavailability of stilbenoids is largely due to rapid and extensive metabolism in the intestine and liver during and after absorption giving rise to a lower level of the free parent compounds [37]. Resveratrol undergoes glucuronidation in the intestine and is mainly absorbed as the glucuronide [38,39]. The remaining resveratrol, absorbed as the aglycone, is further metabolized in the liver to sulfates and glucuronides [40]. High levels of stilbene metabolites such as sulfates and glucuronides have been detected in the plasma and tissues after oral administration [40]. Despite the relatively low bioavailability of parent resveratrol, many studies have demonstrated its biological activities in vivo in various animal studies. Pharmacokinetic studies showed high levels of stilbene metabolites (sulfates and glucuronides) in plasma [41]. Therefore some argue that these metabolites may act as reservoirs for the stilbenoids either by direct action of metabolites [42] or via enterohepatic recycling [31,41]. Stilbenoids are mainly excreted as the metabolites in the urine (renal) and faeces (non-renal). Non-renal routes seem to predominate renal routes of elimination for resveratrol [31] and pterostilbene [32], suggesting as important role for enterohepatic cycling. Thus, biological effects may still be attained with low circulating levels of the parent stilbenoid compounds. 

### 2.3. Stability

In terms of stability, stilbenoids are sensitive to heat, air, light and oxidative enzymes. Specifically, *trans* to *cis* isomerization of resveratrol occurs on exposure to both ultraviolet and visible light [43]. The *trans*- form is known to be the more active form of resveratrol [44]. Furthermore, resveratrol may be degraded by oxidation under certain conditions, for example in the presence of sodium bicarbonate [45,46]. Complexation with cyclodextrin improves both solubility and photostability [25]. Resveratrol was more stable in human plasma than in organic solvents. This is possibly due to binding with plasma proteins such as albumin [47]. The solubility and stability of resveratrol and pterostilbene can also be improved by incorporation into liposomes [26].

### 2.4. Safety

Toxicological data show that at low doses, resveratrol is well-tolerated in humans, although safety information following long-term administration is lacking [48]. Furthermore, administration of pterostilbene in a clinical trial at a dose of 125 mg twice daily for 6–8 weeks was found to be safe and did not evoke any remarkable adverse reactions [49]. The safety of gnetol, as a relatively nascent molecule in research, has not been evaluated in humans. Therefore, additional randomized clinical studies with larger population samples and longer follow-up periods are important to further ascertain the safety of resveratrol and other stilbenoids.

## 3. Cardioprotective Effects of Stilbenoids

Interest in the cardioprotective effects of resveratrol was initially stimulated by observation of the French paradox, in which mortality due to coronary heart disease was significantly reduced among the southwestern French population despite deleterious risk factors such as high intake of dietary cholesterol, saturated fat and smoking [50]. A WHO study, Worldwide Monitoring System for Cardiovascular Diseases (CVD), revealed lower mortality rates for ischemic heart disease in France compared to other developed countries such as the United Kingdom and United States [51]. Cardioprotection was observed despite the presence of similar risk factors for coronary heart disease such as high blood pressure, high body mass index, and high cholesterol [51]. 

The first attempt to explain the French paradox was by Renaud in 1992 in an epidemiological review [52]. Consumption of wine was negatively correlated with CHD mortality [51]. Since there is relatively high wine consumption among the French population, Renaud postulated that the French paradox might be due to the ability of wine consumption to negate the deleterious effects of dairy fat consumption [52]. Thus, phenolic constituents of red wine such as resveratrol garnered attention of biomedical researchers. Many studies have shown the beneficial effects of stilbenoids on vascular function, platelet biology, atherosclerosis, oxidative stress, cardiac hypertrophy and ischemic-reperfusion injury as discussed below. Of the stilbenoids reviewed here, resveratrol is the most widely studied, followed by pterostilbene, piceatannol and gnetol. The cardioprotective effects of resveratrol have been recently reviewed by Zoedoky et al. [53]. 

### 3.1. Vascular Compliance and Blood Pressure 

Resveratrol (2.5 mg/kg/day for 10 weeks) increased mesenteric small artery compliance and reduced wall stiffness in normotensive Wistar–Kyoto (WKY) rats. Resveratrol treatment also attenuated arterial compliance in the spontaneously hypertensive rats (SHR), at least in part through inhibitory actions on pro-growth extracellular signal-regulated kinase (ERK) signaling [54]. The effect of resveratrol on blood pressure in animal models is dose dependent. Resveratrol had no effects on blood pressure at low doses (2.5 mg/kg/day for 10 weeks) [55], whereas administration of higher doses of resveratrol such as 200 mg/kg/day for 4 weeks [56] reduced systolic blood pressure in SHR. Combination of low dose resveratrol (2.5 mg/kg/day) with hydralazine (25 mg/kg/day) was also more effective than resveratrol or hydralazine alone in reducing blood pressure in SHR [57]. Thus, resveratrol may be useful as an adjunct or supplement to current therapies.

Likewise, in humans, a meta-analysis of six randomized control trials showed that resveratrol consumption at a dose of 150 mg/day, but not lower doses, reduced systolic blood pressure. Neither low dose nor high dose resveratrol reduced diastolic blood pressure [58]. To date, there are few studies about the effect of pterostilbene on blood pressure in humans. One randomized double-blinded placebo-controlled trial indicated that high dose pterostilbene (125 mg twice daily) reduced both systolic and diastolic blood pressure while a lower dose of 50 mg twice daily did not [59]. 

### 3.2. Platelet Biology

Among other factors, activation of platelet aggregation is a major contributor to the development of atherothrombosis. In response to rupture of an unstable atherosclerotic plaque, platelets are activated, resulting in thrombus formation [60]. The detached plaque and the resulting thrombus may enter the systemic circulation and cause occlusion of blood vessels, thereby limiting blood supply to organs such as the heart (myocardial infarction) or the brain (stroke). Thus, inhibition of platelet activity is an important strategy to prevent such thrombotic events [61,62].

Resveratrol inhibited platelet aggregation in both animal and human studies [63,64,65]. Furthermore, resveratrol (50 µg/mL) inhibits platelet aggregation induced by collagen, epinephrine, and thromboxane in vitro, and these effects may be attributable to suppression of cyclooxygenase (COX)-1 in the arachidonic acid pathway [66]. Other mechanisms that may be involved include inhibition of the MAPK pathway, activation of the nitric oxide/cGMP pathway [67] and inhibition of phosphoinositide signaling [64].

Similar to resveratrol, pterostilbene also has a strong inhibitory action on platelet aggregation, and stimulates nitric oxide production in platelets [68]. Furthermore gnetol, along with other monomeric stilbenoids (*trans*-resveratrol and isorhapotigenin) extracted from *G. macrostachyum*, inhibited arachidonic acid-induced platelet aggregation [69]. However, gnetol did not inhibit platelet aggregation induced by thrombin [69]. In this study, gnetol also inhibited platelet-collagen adhesion in a dose-dependent manner, although to a lesser extent compared to resveratrol. In addition, in an in vitro ELISA assay reported by Remsberg et al., gnetol inhibited COX with a stronger inhibitory action on COX-1 compared to COX-2 [30].

### 3.3. Ischemia-Reperfusion Injury

Resveratrol (100 µM) attenuates ischemia-reperfusion injury in neonatal cardiomyocytes exposed to 2-h simulated ischemia and 4-h simulated reperfusion possibly by decreasing intracellular calcium, preventing apoptosis and enhancing activities of reactive oxygen species (ROS) scavenging enzymes such as superoxide dismutase (SOD) [70,71]. Other mechanisms that might confer anti-oxidant effects of resveratrol reportedly include modulation of the mitochondrial membrane permeability transition pore (mPTP) [72], activation of adenosine monophosphate (AMP)-activated protein kinase (AMPK) [73], and induction of nitric oxide synthase (NOS) [74,75,76]. 

In cardiomyocytes, pterostilbene protects against hypoxia-reoxygenation injury via activation and up-regulation of sirtuin 1 (SIRT1) [77]. SIRT1 is a NAD+-dependent protein deacetylase that, upon activation, activates peroxisome proliferator-activated receptor gamma coactivator-1-alpha (PGC-1α) via deacetylation and thus improves mitochondrial function and oxidative capacity [78]. Here, pre-treatment of H9c2 cells with splitomycin, a SIRT1 inhibitor, abolished the protective effects of pterostilbene [77]. In an animal model of ischemia-reperfusion injury, pterostilbene improved cardiac function and reduced markers of oxidative stress and inflammation such as tumor necrosis factor-alpha (TNF-α), interleukin-1-beta (IL-1β) and myeloperoxidase activity [79]. These data are consistent with results from other groups that showed protective effects of pterostilbene on myocardial ischemia-reperfusion injury via inhibition of apoptosis and attenuation of inflammatory markers in rats [80,81]. Pterostilbene improved cardiac function and decreased myocardial infarct size in a rat model of ischemia-reperfusion injury [80]. Here treatment with pterostilbene reduced expression of TNF-α and IL-1β, thus reducing cardiac inflammation. Furthermore, pterostilbene increased Bcl-2 expression while decreasing Bax expression [80]. 

A study by Hung and colleagues investigated the beneficial effects of astringin, a 3-*β*-d-glucoside of piceatannol, in the setting of ischemia reperfusion (I/R) injury in a rat model [82]. Pre-treatment with astringin significantly reduced the incidence and duration of both ventricular tachycardia and fibrillation [82]. Varying doses of astringin were also associated with decreased mortality rates, increased nitric oxide (NO) and decreased lactate dehydrogenase (LDH) levels, supporting its role as a potent antiarrhythmic and cardioprotective agent [82] 

### 3.4. Atherosclerosis

There are two major types of lipoprotein that function as carriers of cholesterol in the systemic circulation: high density lipoprotein (HDL) and low-density lipoproteins (LDL). High levels of HDL in the circulatory system are considered “good” while high levels of LDL “bad”. Due to their low molecular weight, a high level of LDL predisposes to lipid accumulation in the arterial wall, leading to atherogenic processes [83]. Furthermore, LDL oxidation plays an important role in atherogenesis. Oxidized LDL promotes accumulation of inflammatory cells such as macrophages, thereby causing build-up of plaques on the vessel wall [84,85]. Therefore, suppression of LDL oxidation is an important anti-atherosclerotic therapeutic target. Flavonoids from grape juice and red wine, for example, inhibit plasma oxidation of LDL in humans [86].

Resveratrol modifies vascular function [54,87], attenuates lipid accumulation [88,89], and modulates gene expression related to lipogenesis and lipolysis [90]. Resveratrol also inhibits oxidized LDL (oxLDL)-induced apoptosis in vascular endothelial cells [91,92]. Similarly, pterostilbene protects human vascular endothelial cells against apoptosis [93]. Pterostilbene induced cytoprotective autophagy in vascular endothelial cells via activation of AMPK and downstream inhibition of mTOR signaling [94]. Here, pterostilbene activated AMPK via upstream calcium/calmodulin-dependent protein kinase kinase beta (CAMKKβ) [94]. Recent studies indicate that pterostilbene attenuates high fat-induced atherosclerosis in mice via suppression of pro-inflammatory cytokines such as transforming growth factor beta (TGFβ), TNFα, IL-1β and IL-6 among others [95]. Pterostilbene inhibits proliferation of vascular smooth muscle cells and progression of cell cycle by regulating Akt kinase [96]. In several notable studies, piceatannol given at variable doses (15–45 mg/kg) resulted in reduced plasma lipopolysaccharides, LDL-cholesterol levels and lipid peroxidation in a murine model [97,98].

Taken together, these research findings suggest protective properties of stilbenoids on the vascular endothelium, which may delay the initiation and progression of atherosclerosis.

### 3.5. Cardiac Hypertrophy

Resveratrol prevents cardiomyocyte and cardiac hypertrophy in isolated NE-treated cardiomyocytes and in the pressure overload model of cardiac hypertrophy [99,100,101]. One possible explanation of anti-hypertrophic actions of resveratrol is via activation of NO-AMPK signaling [99]. Resveratrol also inhibited hypertrophy induced by pressure overload (concentric hypertrophy) but not volume overload (eccentric hypertrophy) [102]. The proposed mechanism for the effect of resveratrol on pressure overload-induced hypertrophy includes alleviation of oxidative stress and increased NO production via upregulation of endothelial nitric oxide synthase (eNOS) [102]. Of note, eNOS level and activity remain unchanged in the volume-overload model of hypertrophy [103], suggesting that unique signaling pathways are involved in pressure-overload versus volume-overload hypertrophy. Therefore, the authors concluded that resveratrol may be used in pressure-overload disease settings such as hypertension and aortic stenosis [102]. Resveratrol also prevented development of hypertrophy in SHR, a genetic model of hypertension and hypertrophy, via the LKB-AMPK-eNOS signaling axis [55,104], and grape powder containing phenols such as resveratrol, anthocyanins and catechins improved cardiac and vascular function in SHR [105]. 

In a recent study, we showed that gnetol and pterostilbene inhibited ET-1-induced hypertrophy in isolated neonatal rat cardiomyocytes via activation of AMPK [106]. A comparative study using equal doses (2.5 mg/kg/day) of gnetol, pterostilbene and resveratrol for 8 weeks did not inhibit left ventricular hypertrophy in the spontaneously hypertensive heart failure (SHHF) rat, which is a model of hypertrophy and heart failure [106]. However, the three stilbenoids improved diastolic function as evidenced by preventing prolongation of iso-volumetric relaxation time, and thus, still exerted some cardioprotection beyond antihypertrophic effects [106].

### 3.6. Cardiovascular Human Studies

Despite the promising effects described above for stilbenoids, the efficacy of specific grape phenols such as resveratrol is yet to be established in humans. For example, administration of resveratrol (75 mg/day for 12 weeks; *n* = 15) to non-obese healthy postmenopausal women did not change metabolic parameters such as insulin sensitivity, mitochondrial function, AMPK signaling and inflammatory markers [107]. The absence of effect of resveratrol on metabolic parameters in healthy subjects is consistent with earlier results obtained in healthy rodents [108]. Thus, we may infer that resveratrol likely exerts its effects only in metabolic disease conditions such as obesity, type II diabetes and dyslipidemia. 

Resveratrol administration for primary and secondary prevention of CVD have shown some promise [109,110,111], but small sample size (*n* = 75) and short follow-up (1 year) limit the clinical relevance. In this study, 75 subjects were randomly divided into 3 groups. One group was administered resveratrol-containing grape extract capsules (8.1 mg/day for the first 6 months and 16.2 mg/day for the next 6 months), another group was given grape extract capsules containing no resveratrol, and the third group was given placebo capsules. All subjects in this study were also on statins and were treated according to the prevailing guidelines for primary prevention of CVD [110]. At the study endpoint, patients treated with resveratrol-containing grape extract showed improved inflammatory and fibrinolytic status compared to placebo and grape-extract only group [110]. Since resveratrol provided additional benefits in patients with high risk of CVD, over and above other phenols in the grape extract, resveratrol may complement current guidelines for the primary prevention of CVD [109,110]. 

Although resveratrol and pterostilbene exerted antihyperlipidemic actions in animal models [112,113], clinical trials in humans do not show reduction in LDL/HDL ratio. In a meta-analysis of randomized control trials, supplementation with resveratrol did not significantly affect lipid parameters such as total cholesterol, LDL, HDL and triglyceride levels [114]. Both high and low doses of pterostilbene increased LDL and had no effect on HDL and triglycerides in a randomized placebo-controlled trial [59]. Administration of grape extract had no effect on LDL levels. 

To determine effects on secondary prevention of CVD, resveratrol-containing grape extract (8.1 mg/day for the first 6 months and 16.2 mg/day for the next 6 months) was administered to patients with stable coronary artery disease in addition to their regular medications and dietary restrictions [111]. Compared to the placebo and grape extract only group, there was an increase in anti-inflammatory adiponectin and a decrease in thrombogenic plasminogen activator inhibitor type 1 (PAI-1) in the group that received resveratrol [111]. Thus, resveratrol may exert its cardioprotective effects by improving anti-inflammatory response and preventing atherothrombotic signaling [111].

## 4. Other Biological Effects of Stilbenoids

### 4.1. Diabetes

Diabetes is a chronic metabolic disease associated with inflammation and oxidative stress. Due to its anti-inflammatory and antioxidant effects, resveratrol can mitigate the development of diabetic complications. Preclinical data show that resveratrol might be beneficial in the management of diabetes by improving insulin resistance, improving defective insulin signaling, and preventing pancreatic beta cell apoptosis and dysfunction [115]. Resveratrol prevents hyperglycemia in diabetic animal models by increasing glucose uptake and translocation of GLUT 4 to the caveolar membrane of diabetic myocardium [116]. Furthermore, resveratrol improved glucose tolerance and reduced the expression of advanced glycated end-products (AGE) receptors in diabetic rat liver and kidney [117,118]. Resveratrol prevents production of ROS and reactive nitrogen species such as superoxide anion (O_2•_^−^), hydroxyl radical (OH^•^), hydrogen peroxide and malondialdehyde (MDA) while increasing levels of antioxidant enzymes such as SOD, catalase and glutathione peroxidase in diabetic animals [119]. Resveratrol also inhibits the pro-inflammatory signaling, nuclear factor ĸB (NFĸB), and reduces the production of inflammatory cytokines such as TNF-α, IL-1β, IL-4, and IL-6 [119]. In addition, resveratrol increased insulin sensitivity, glucose tolerance and mitochondrial biogenesis in an AMPK-dependent manner [120]. In this study, resveratrol was unable to elicit the same effect in AMPK-deficient mice suggesting the role of AMPK in the metabolic actions of resveratrol [120].

While some human studies have shown the benefits of resveratrol on glycemic control, other studies did not demonstrate significant effects. Administration of resveratrol (250 mg/day for 3 months) in addition to either metformin or glibenclamide improved glycemic parameters in type 2 diabetes patients, as compared to metformin or glibenclamide alone [121]. Movahed and colleagues also reported that resveratrol (1 g/day for 45 days) reduced fasting blood sugar, HbA1c and systolic blood pressure [122]. Indeed, a much lower dose of resveratrol (5 mg/day for 28 days) reduced hemoglobin A1c (HbA1c), systolic blood pressure and improved insulin sensitivity but did not affect the homeostatic model of assessment of insulin resistance (HOMA-β).

In contrast, in a randomized control trial by Thazhath et al., administration of 500 mg of resveratrol twice daily for 5 weeks in diet-controlled type 2 diabetes did not significantly improve glycemic control [123]. There was no difference between the fasting glucose level, postprandial glucose level, HbA1c, gastric emptying and glucagon-like peptide 1 secretion in the resveratrol-treated versus the placebo group. Similarly, resveratrol treatment for 6 months did not improve metabolic parameters in type 2 diabetic patients [124]. Therefore, the effect of resveratrol on diabetes in human, if any, is not fully understood.

Pterostilbene improves glycemic control in insulin-resistant obese rats by increasing hepatic glucokinase activity and increasing skeletal muscle glucose uptake [125]. In vitro studies also indicate that pterostilbene protected pancreatic beta cells against oxidative stress and apoptosis [126]. Antihyperglycemic properties of pterostilbene along with other phenolic constituents of *Pterocarpus marsupium* have been reported [127,128]. Whereas pterostilbene has been shown to be beneficial in animal models of diabetes and metabolic disorders, human data are still limited. The effect of pterostilbene on human type 2 diabetes is yet to be explored. Administration of blueberry (*Vaccinium myrtillus*) and sea buckthorn (*Hippophae rhamnoides*) extract to children with type 1 diabetes for two months elicited a reduction in HBA1c levels and an increase in SOD and glutathione peroxidase levels [129]. Since pterostilbene has been isolated from *Vaccinium myrtillus* [130], this effect may be due to the presence of pterostilbene alongside other bioactive compounds in the extract.

### 4.2. Neurodegeneration

The neuroprotective effects of stilbenoids are mostly due to their anti-oxidant and anti-inflammatory properties [131,132,133,134]. Neurodegenerative disorders such as Parkinson’s and Alzheimer’s diseases are associated with oxidative stress and mitochondrial dysfunction leading to loss of function and death of neurons [135]. Resveratrol protects neurons against ROS and improved motor co-ordination in 1-methyl-4-phenyl-1,2,3,6-tetrahydropyridine (MPTP)-induced parkinsonism in mice by scavenging hydroxyl radicals [136]. Resveratrol also protected against lipopolysaccharide-induced dopaminergic neurodegeneration via inhibition of microglial activation and NFĸB signaling in the microglial [137]. Alzheimer’s disease is characterized by development of plaques made up of amyloid-beta protein in the hippocampus and cerebral cortex. Aggregation of amyloid-beta plays a key role in the pathogenesis of Alzheimer’s disease [138]. There is also evidence that amyloid-beta contributes to oxidative damage in the neurons by inducing lipid peroxidation, protein oxidation and DNA oxidation [139]. Resveratrol holds therapeutic potential in the treatment of Alzheimer’s disease due to its ability to reduce amyloid plaques in brain. In a study by Marambaud et al., although resveratrol did not inhibit the production of amyloid-beta, it promoted proteasome-dependent degradation of amyloid-beta [140]. 

Pre-treatment with resveratrol protects against cerebral ischemia-reperfusion injury in rats [141]. Levels of nuclear factor erythroid 2-related factor (Nrf2) and heme oxygenase-1 (HO-1) were upregulated in the resveratrol-treated group, suggesting reduction in oxidative damage during cerebral ischemia [141]. Infarct volume and brain water content were reduced and neurological scores were improved by resveratrol pre-treatment. Resveratrol protected against neuronal death in a rat model of global cerebral ischemia via activation of PI3K-Akt signaling and downregulation of glycogen synthase kinase-3β (GSK-3β) and cAMP response element-binding protein (CREB) [142]. Apart from effects on cerebral ischemia, resveratrol improved cognition in an animal model of vascular dementia [143]. Here, vascular dementia was induced by bilateral occlusion of the common carotid arteries for 8 to 12 weeks. Treatment with resveratrol improved learning and memory scores. The lipid peroxidation product, malondialdehyde, was reduced while levels of antioxidant enzymes such as SOD and glutathione were increased in the hippocampus and cerebral cortex of the resveratrol-treated group [143]. This suggests the antioxidant role of resveratrol in the neuroprotective effects. 

Although reports on the effects of pterostilbene on specific animal models of Alzheimer and Parkinsonism are limited, effects in cell culture models of neurotoxicity have been reported. Pterostilbene exerts neuroprotective effects against high glucose-induced injury in neuroblastoma cells [131]. Here, pterostilbene prevented cell death and generation of ROS in a dose-dependent manner. Pterostilbene also increased activities of mitochondrial complexes I and III, mitochondrial cytochrome C, and mitochondrial membrane potential. In addition, the levels of Nrf2, HO-1 and glutathione S-transferase (GST) were elevated with pterostilbene treatment, indicating protection against neuronal oxidative stress [131]. Similarly, pterostilbene ameliorates glutamate-induced neuronal oxidative stress via Nrf2 signaling [132]. In a recent study, streptozotocin-induced memory deficit in Sprague-Dawley (SD) rats was improved by pterostilbene treatment [144]. Pterostilbene also improved cholinergic transmission via inhibition of cholinesterases [144]. A study investigating the neuroprotective effect of oxyresveratrol in rat cortical neurons demonstrated that the hydroxystilbene prevented Aβ (25–35)-induced neuronal cell damage by preventing increase in cytosolic [Ca^2+^] levels, inhibiting glutamate release and reducing ROS generation [145]. 

In a separate study, Andrabi et al. demonstrated the neuroprotective effect of oxyresveratrol in a transient murine middle cerebral artery occlusion (MCAO) model [146]. At various doses of 10 or 20 mg/kg, oxyresveratrol significantly reduced the brain infarct volume of MCAO rats, improved neurological deficits caused by I/R injury, and inhibited cytochrome c release as well as caspase-3 activation in MCAO rats. These findings indicate the potent neuroprotective effect of oxyresveratrol and its potential use for the treatment of stroke [146].

Studies on the effect of gnetol on the nervous system are scarce. There is one report that gnetol reversibly and competitively inhibits butyryl cholinesterase, and this may be applicable in the treatment of Alzheimer disease [147]. 

In a randomized placebo-controlled clinical trial, resveratrol, at a dose of 500 mg per day gradually escalated to 1000 mg twice daily for 52 weeks, was found to be safe and well-tolerated in Alzheimer disease patients [148]. In this study, resveratrol prevented reduction of cerebrospinal fluid and plasma levels of amyloid-beta 40 (the most abundant amyloid beta isoform) compared to the placebo group, but did not affect several other important Alzheimer disease biomarkers [148]. Thus, the results of this study did not clearly indicate benefit of resveratrol in Alzheimer disease. In contrast, supplementation with 200 mg/day resveratrol and 320 mg/day quercetin (to improve bioavailability of resveratrol) for 26 weeks improved memory performance in healthy overweight elderly subjects [149]. A more recent clinical trial also indicated that resveratrol improves cognition, mood and cerebrovascular function in postmenopausal women when administered at a dose of 75 mg twice daily for 14 weeks [150]. Resveratrol improved cerebral blood flow by dilating cerebral blood vessels and thus improved cognitive performance in type 2 diabetic patients [151,152]. In conclusion, results of human studies show that resveratrol improves memory function and cognition in healthy subjects and diabetic patients with sub-clinical cognitive impairment but not in Alzheimer disease per se. The effects of pterostilbene and gnetol on memory and cognition are yet to be studied in humans. 

### 4.3. Obesity 

Obesity is characterized by excessive adipose tissue caused by increased caloric intake and/or inadequate energy expenditure. As defined, body mass index greater than 30 and waist circumference greater than 88 cm and 94 cm in women and men respectively [153] is one of the major risk factors for the development of CVD. Resveratrol inhibits adipogenesis, attenuates lipid accumulation and increases lipolysis in mature adipocytes [154]. The lifespan of mice fed high caloric (60% fat) diets supplemented with 0.04% resveratrol was lengthened [155]. Furthermore, administration of 150 mg/day *trans*-resveratrol over 30 days mimics the effect of caloric restriction in obese humans and thus may help to control obesity and other metabolic syndromes [156]. Similarly, resveratrol delayed age-related abnormalities, albeit without significant effect on longevity in non-obese mice [157]. This indicates that resveratrol may help counter the adverse metabolic effects of obesity. In non-human primates, resveratrol was shown to suppress body mass gain, increase metabolic rates and total energy expenditure [158,159]. 

Since resveratrol activates AMPK, and AMPK is involved in energy balance regulation and mitochondrial biogenesis, AMPK was proposed as a possible mechanism by which resveratrol protects against metabolic dysfunction [120]. In AMPK-deficient mice, resveratrol failed to improve insulin sensitivity possibly due to non-effect on mitochondrial content and fatty acid oxidation in skeletal muscles. This caused a build-up of lipids that may inhibit insulin action [120]. Furthermore, resveratrol activates sirtuins [160]. In high fat-fed mice, resveratrol improved glucose tolerance by enhancing sensitivity to insulin, and protected the mice against development of obesity [161]. This beneficial effect of resveratrol was lost when the acetylation sites of PGC-1α were mutated or when SIRT expression was disrupted using SIRT-deficient mice, thus indicating the role of PGC-1α /SIRT signaling [161].

Similar to resveratrol, pterostilbene exhibits anti-obesity properties. Pterostilbene reduces fat accumulation in adipose tissue by inhibiting lipogenesis, while enhancing fatty acid oxidation in the liver [162]. Pterostilbene also increased AMPK and acetyl-coA carboxylase activities in adipose tissue. Furthermore, in Zucker *fa*/*fa* rats, pterostilbene increased thermogenesis in brown adipose tissue by increasing the gene expression and translation of uncoupling protein 1 (UCP-1), a key mediator of thermogenesis [163]. Another recent study showed that pterostilbene reduced accumulation of abdominal white adipose tissue, increased fat metabolism and suppressed lipogenesis in obese rats [164]. Here, mRNA levels of UCP1 were also increased while mRNA levels of fatty acid synthase and leptin were reduced [164]. While effects on obesity have been reported for both resveratrol and pterostilbene as discussed above, the effect of gnetol on obesity is yet to be explored.

Despite promising effects of resveratrol on obesity in animal models, a systematic review of nine randomized controlled trials on the effects of resveratrol on obesity in humans showed limited evidence for the use of resveratrol in obesity and weight management [165]. Most studies did not find a reduction in body weight after treatment with resveratrol for a period of 4 to 12 weeks in obese and non-obese subjects [107,156,166,167]. The only study that reported a clear beneficial effect on body weight administered resveratrol 500 mg three times per day for 12 weeks in obese subjects with metabolic syndrome [168]. Thus, it may be concluded that a period of at least 12 weeks may be required for resveratrol to elicit anti-obesity effects in obese humans [169]. Further human studies are required to confirm the effects of resveratrol, and the results of ongoing clinical trials may provide more information in this regard.

### 4.4. Cancer Treatment and Prevention

Interventions to prevent and/or treat cancer may occur at specific stages of carcinogenesis such as initiation, promotion and progression. Stilbenes block metabolic activation of pro-carcinogens by inhibiting specific isoforms of cytochrome P450 (CYP) enzymes and thus prevented the initiation of carcinogenesis in cultured human tumor cells [170,171]. Resveratrol may also protect against cancer by inducing phase II metabolism of carcinogens, thereby enhancing their elimination from the body [172]. For instance, resveratrol induced activity of metabolizing enzymes such as uridine-5-diphospho (UDP) glucuronyltransferase and NADPH:quinone oxidoreductase in mouse epidermis [173]. Resveratrol may prevent the progression of cancer by suppressing actions of transcription and growth factors, including p53, FoxO, and ATF3, which are involved in the initiation and promotion of cancer in cell culture studies [174]. Furthermore, resveratrol and pterostilbene prevent proliferation and induce apoptosis in various cancer types including breast, prostate, pancreatic, liver and colorectal cancer (recently reviewed by Carter et al. [175,176]). Mechanisms that have been implicated in the anticancer properties of resveratrol and pterostilbene include induction of apoptosis, inhibition of proliferation, cell cycle arrest and inhibition of angiogenesis [177,178]. 

Pterostilbene exerts more potent inhibitory actions on human colon cancer cells than resveratrol due to its higher lipophilicity [179]. Hagiwara and colleagues demonstrated the ability of pterostilbene (50 μM) to promote the expression of tumor-suppressive miRs and argonaute2 (Ago2), a central RNA interference component, in breast cancer cells [180]. Piceatannol, an analog of resveratrol, suppressed various cancer types. Piceatannol suppressed proliferation and invasion of AH109A hepatoma cells via cell cycle arrest, apoptosis and anti-oxidation [181]. In addition, two separate groups demonstrated that through the inhibition of matrix metallopeptidase (MMP)-9, various doses of piceatannol reduced the metastasis of prostate and breast cancer cells [182,183]. Finally, in a recent study, gnetol was shown to possess anti-cancer potency through the inhibition of histone deacetylases and cytochrome enzymes (CYP2C9 and CYP3A4) [30], which serves as a prospective area for further investigation. 

Although data from animal studies are promising, there is limited human clinical data to support the use of stilbenoids in the treatment and management of cancer. Thus, more studies are needed in this area before resveratrol and other stilbenoids can be considered for use in the setting of human cancer treatment and prevention.

### 4.5. Depigmentation

Melanin, a pigment which protects the skin against harmful effects of ultra-violet radiation, is produced by melanocytes in the process of melanogenesis [184]. Overproduction of melanin in some acquired hyperpigmentation disorders may cause skin concerns [185]. Furthermore, deregulation of melanogenesis has been associated with more aggressive progression of melanotic melanomas which may be due to increased production of cytotoxic and immunosuppressive intermediates, such as quinone, semiquinone and other ROS [186,187]. Thus, inhibition of melanogenesis may serve as an adjuvant therapy in the treatment of melanomas [188]. Melanogenesis is a multistep process which begins with conversion of l-tyrosine to l-3,4-dihydroxyphenylalanine (l-DOPA), the rate-limiting step catalyzed by the enzyme tyrosinase [184]. Gnetol inhibited melanin production in murine B16 melanoma cells via inhibition of tyrosinase [12]. 

Similarly, resveratrol inhibited ultraviolet B-induced hyperpigmentation in guinea pig skin [189]. Here, resveratrol also downregulated melanogenesis-related proteins such as tyrosinase, tyrosinase related protein (TRYP) 1, TRYP2 and microphthalmia-associated transcription factor (MITF) in melanoma cells [189]. There is evidence to show that pterostilbene is a more potent inhibitor of melanogenesis than resveratrol in α-melanocyte stimulating hormone (MSH)-stimulated B16/F10 melanoma cells [190]. Moreover, pterostilbene inhibited tyrosinase enzyme activity in a dose-dependent manner [190]. A study by Kim and colleagues investigated oxyresveratrol and demonstrated a potent inhibitory effect of 1.2 μM (IC_50_ value) on mushroom tyrosinase activity. Oxyresveratrol depigmentation is believed to exert its effects through reversible inhibition of tyrosinase activity and is dependent on the number and position of hydroxy substituents the compound contains [191].

The antimelanogenetic properties of piceatannol have also been investigated, with results indicating that piceatannol down-regulates melanin content and has a stronger anti-tyrosinase activity than both kojic acid, a chelation agent with moderate anti-pigment properties, and resveratrol [192]. The actions of piceatannol are likely attributed to anti-oxidative actions along with the ability to suppress reactive species generation while increasing the glutathione/oxidized glutathione ratio [192]. The ability of stilbenoids to inhibit melanin synthesis qualifies them as suitable potential candidates for use in the cosmetic industry to treat acquired hyperpigmentation disorders and possibly as adjuvants in the treatment of melanotic melanomas.

## 5. Conclusions

In summary, stilbenoids exert various biological effects such as cardioprotection, neuroprotection, anti-diabetic properties, depigmentation, anti-inflammation, cancer prevention and treatment. These effects are thought to be mediated by several universal signaling pathways. For instance, stilbenoids inhibited platelet aggregation possibly through inhibition of COX-1. Stilbenoids also reduced markers of oxidative stress and inflammation (TNF-α, IL-1β) in various models of ischemia-reperfusion injury and inhibited cardiomyocyte hypertrophy via activation of AMPK. In addition to the beneficial effects seen in the cardiovascular setting, stilbenoids improved insulin resistance and glucose tolerance in animal models of diabetes, induced apoptosis and inhibited proliferation of various cancer cell lines, and inhibited melanogenesis via downregulation of tyrosinase and other related proteins. The results presented in this review cover a myriad of models, from cell culture to animal studies as well as human studies (Table 2). Although positive results were obtained in most cell culture and animal studies, further human studies are needed to substantiate the beneficial effects of stilbenoids. Resveratrol remains the most widely studied stilbenoid compound. However, there is limited information regarding the potential beneficial properties of less common stilbenoids. Therefore, further research is warranted to evaluate the beneficial health effects of less known stilbenoid compounds. 

## Figures and Tables

**Table 1:** Half-lives and oral bioavailabilities of stilbenoids after oral administration in rats.

Stilbenoid	Oral Dose (mg/kg)	Half Life (h)	Oral Bioavailability (Rats) (%)
resveratrol	50	1.48 [31]	29.8 [31]
pterostilbene	20	1.73 [32]	80 [29]
gnetol	100	4.2 [30]	6.59 [30]
piceatannol	10	4.23 [33]	50.7 [34]
oxyresveratrol	24.4	–	9.13 [35]

**Table 2:** Summary of biological effects of stilbenoids.

Study	Model	Stilbenoid	Dose	Result
Cardiovascular/Blood Pressure				
Behbahani	WKY rat	resveratrol	2.5 mg/kg per day (for 10 weeks)	increased compliance and reduced wall stiffness in mesenteric small arteries
Thandapilly et al. [55]	SHR	resveratrol	2.5 mg/kg per day (for 10 weeks)	prevention of developed concentric hypertrophy, systolic/diastolic dysfunction; no effect on blood pressure (BP)
Li et al. [56]	SHR	resveratrol	200 mg/kg (for 4 weeks)	increased endothelial NO production; reduced BP
Riche et al. [59]	human	pterostilbene	125 mg twice daily (for 6–8 weeks)	increased LDL cholesterol and reduced BP
Tome-Carneiro et al. [110]	human, stable coronary artery disease	grape extract + resveratrol	8.1 mg/day (6 months); then 16.2 mg/day (6 months)	increased anti-inflammatory serum adiponectin, decreased thrombogenic PAI-1
**Platelet biology**				
Olas et al. [63]	in vitro	resveratrol	25–100 μg/mL	inhibition of adhesion of platelets to fibrinogen/collagen
I/R Injury				
Yu et al. [79]	SD rat (30 min ischemia; 3 h reperfusion)	pterostilbene	10 mg/kg	reduced superoxide generation, MDA; increased SOD; reduced myocardial infarction and apoptosis
Hung et al. [82]	SD rat	piceatannol	2.5 × 10^−4^ g/kg	reduced incidence and duration of ventricular tachycardia, ventricular fibrillation; prevention of mortality, increased NO and decreased LDH levels
**Diabetes**				
Um et al. [120]	AMPK subunit (α1/α2) deficient mice	resveratrol	400 mg/kg per day (12 weeks)	AMPK dependent: increased insulin sensitivity, glucose tolerance and mitochondrial biogenesis
Bhatt et al. [121]	humans (with type II diabetis mellitus)	resveratrol	250 mg/day for 3 months	improved HbA1c, systolic BP, total cholesterol and total protein
Gomez-Zorita et al. [125]	diabetic rat (induced by obesogenic diet)	pterostilbene	15–30 mg/day for 6 weeks	improved glycaemic control due to increased hepatic glucokinase activity and skeletal muscle glucose uptake
Nemes-Nagy et al. [129]	human (children with T1DM)	blueberry and sea buckthorn concentrate	3 × 1 comprimates per day for 2 months	increased SOD activity, decreased levels of glycated hemoglobin and increased C peptide concentration
**Neurodegeneration**				
Ren et al. [141]	SD rat	resveratrol	15–30 mg/kg for 7 days	Reduced cerebral infarct volume, decreased MDA levels, restored SOD activity, increased Nrf2 and HO-1 and reduced caspase-3 expression
Ma et al. [143]	rat model (vascular dementia)	resveratrol	25 mg/kg per day	decreased malonyldialdehyde levels; increased SOD activity and glutathione levels; improved learning and memory ability
Naik et al. [144]	SD rat (streptozotocin-induced memory deficit)	pterostilbene	10–50 mg/kg per day for 13 days	improved memory and cognition; improved brain antioxidants [catalase, SOD, glutathione (GSH)]; improved cholinergic transmission.
Ban et al. [145]	SD rat	oxyresveratrol	10 μM	inhibition of Aβ-induced neuronal cell death, elevation of cytosolic [Ca] and ROS generation
Evans et al. [150]	human (postmenopausal women)	resveratrol	75 mg twice per day for 14 weeks	improved memory, mood and overall cognitive performance
**Obesity**				
Timmers et al. [156]	Human (obese)	resveratrol	150 mg/day for 30 days	reduced sleeping and resting metabolic rate; in muscle, activated AMPK, increased SIRT1 and PGC-1α protein levels; decreased systolic BP and improved HOMA index
Aguirre et al. [163]	Zucker (*fa*/*fa*) rat model	pterostilbene	15–30 mg/kg per day for 6 weeks	increased thermogenic and oxidative capacity of brown adipose tissue
**Cancer**				
Nutakul et al. [179]	human colon cancer cells	resveratrol and pterostilbene	0–100 μM for between 30 min to 10 days	pterostilbene: more potent inhibitor of colony formation, stronger apoptosis-inducing effects, and 2–4-fold higher intracellular pterostilbene levels than resveratrol
Jayasooriya et al. [178]	human prostate cancer cells	piceatannol	0–40 μM for 24 h	inhibition of TNF-α-induced invasion of cancer cells through suppression of MMP-9 activation via the Akt-mediated NF-ĸB pathway
Remsberg et al. [30]	SD rats	gnetol	10–100 mg/kg per day, for 0–72 h	reductions in cell viability in cancer cell lines (i.e., colorectal cancer); activities in COX-1, COX-2, histone deacetylase and decreased inflammation
**Pigmentation**				
Lee et al. [189]	male guinea pig model	resveratrol	dissolved in ethanol/propylene glycol (3:7, *v*/*v*)	reduced expression of melanogenesis-related proteins; decreased hyperpigmentation in ultraviolet B-stimulated skin
Yoon et al. [190]	B16/F10 murine melanoma cells	pterostilbene and resveratrol trimethyl ether (RTE)	10 μM for 48 h	inhibition of α-MSH-induced melanin synthesis, stronger downregulation of tyrosinase protein expression and α-MSH stimulated protein than RTE
Yokozawa et al. [192]	B16/F10 melanoma cells	piceatannol	0–400 μM for 24 h	greater antityrosinase activity than kojic and resveratrol; down-regulation of melanin content, suppressed ROS generation and enhanced GSH/GSSG ratio

## Abbreviations

AGE	Advanced glycated end-products
Akt	Protein kinase B
AMP	Adenosine monophosphate
AMPK	AMP-activated protein kinase
CAMKKβ	Calcium/calmodulin-dependent protein kinase kinase beta
cAMP	Cyclic adenosine monophosphate
cGMP	Cyclic guanosine monophosphate
CHD	Coronary heart disease
COX	Cyclooxygenase
CREB	cAMP response element-binding protein
CVD	Cardiovascular disease
CYP	Cytochrome P450
ELISA	Enzyme-linked immunosorbent assay
eNOS	Endothelial nitric oxide synthase
ERK	Extracellular signal-regulated kinase
GLUT4	Glucose transporter 4
GSH	Glutathione
GSK-3β	Glycogen synthase kinase 3 beta
GSSH	Glutathione disulfide
GST	Glutathione S-transferase
HbA1c	Hemoglobin A1c
HDL	High density lipoprotein
HO-1	Heme oxygenase-1
HPLC	High performance liquid chromatography
IL	Interleukin
I/R	Ischemia-Reperfusion
LDH	Lactate dehydrogenase
l-DOPA	l-dihydroxyphenylalanine
LDL	Low density lipoprotein
LKB	Liver kinase B
MAPK	Mitogen-activated protein kinase
MCAO	Middle cerebral artery occlusion
MITF	Microphthalmia-associated transcription factor
MMP	Matrix metallopeptidase
mPTP	Membrane permeability transition pore
mRNA	Messenger ribonucleic acid
MSH	Melanocyte Stimulating Hormone
mTOR	Mammalian target of rapamycin
NAD	Nicotinamide adenine dinucleotide
NE	Norepinephrine
NFκB	Nuclear factor kappa B
NO	Nitric oxide
NOS	Nitric oxide synthase
O_2_^−^	Superoxide anion
^•^OH	Hydroxyl radical
PAI	Plasminogen activator inhibitor
PGC-1α	Peroxisome proliferator-activated receptor gamma coactivator-1-alpha
ROS	Reactive oxygen species
SD	Sprague-Dawley
SHR	Spontaneously hypertensive rat
SIRT1	Sirtuin 1
SOD	Superoxide dismutase
TGF-β	Transforming growth factor-beta
TNF-α	Tumor necrosis factor-alpha
TRYP	Tyrosinase-related protein
UCP	Uncoupling protein
UDP	Uridine diphosphate
WHO	World Health Organization
WKY	Wistar-Kyoto
